# Retrospective determination of exposure temperature of standing trees during wildfires with solid-state NMR

**DOI:** 10.1038/s41598-024-63754-w

**Published:** 2024-06-04

**Authors:** Karl Kutzer, Martina Meincken

**Affiliations:** Department of Forest and Wood Science, Stellenbosch University, Stellenbosch, South Africa

**Keywords:** Nuclear magnetic resonance, Wildfires, Pine, Eucalyptus, Temperature prediction, Chemical degradation, Plantation forestry, Woods water environment, Thermal stability, Natural hazards, Forestry, Materials science, Techniques and instrumentation

## Abstract

Timber plantations across the world are suffering from the effects of increasingly frequent wildfires, which potentially degrade the wood of affected trees, depending on the exposure temperature and time. However, it is rather complicated to determine the exact temperature of the fire, or the temperature to which the wood was exposed. This study aimed to determine the exposure temperature of wood retrospectively through solid-state NMR analysis. Models were developed from softwood and hardwood samples exposed to defined temperatures, which successfully linked the NMR signal to the exposure temperature. Various fit equations were developed to link the half-width or peak area of the NMR signal to the exposure temperatures. Hard- and softwoods displayed noticeable differences: a linear function best described the half-width in the higher temperature region for Pine and Eucalyptus, whereas a parabolic function for the peak area of Eucalyptus yielded the best correlation to the entire temperature range. This non-destructive and direct method offers a valuable evaluation method to determine, if wood in burnt trees is degraded and can be processed. An informed choice can be made on the decision to use, or discard burnt wood.

## Introduction

The world is under increasing threat from wildfires due to external factors, such as global warming and increasing human population. According to Pausas and Keeley^1^, human factors promote these changes in fire regime and the combination of factors such as ignition, suitable fire weather and drought lead to increasing occurrence of wildfires.

Plantations across Southern Africa are suffering from the increasing occurrence of wildfires. Wildfires are nothing new to the country and play a vital role in fynbos regeneration and growth, especially in the Western Cape, but they are a major risk factor for the forest sector. They occur according to seasonal patterns at natural cycles^2^. However, with the impact of global warming these patterns are increasingly disturbed. South Africa lost over 80,000 ha of plantation forestry in 2007 and these numbers increased in the following years. In the winter of 2017, wildfires burned about 15 000 ha over a four-day period around Knysna in the Southern Cape of South Africa. This resulted in loss of life, the destruction of over 800 buildings and the destruction of over 5000 ha of commercial forestry plantations with the estimated financial loss estimated to be around R3 billion^3^.

The exposure to high temperatures leads to wood degradation and subsequent loss of material, which makes it difficult to determine which trees are still usable and which should be discarded. However, without the knowledge of the exposure temperature that the trees encountered, it is impossible to estimate the amount of wood degradation that occurred.

In a previous pilot study^4^ a new method was developed in an attempt to predict the exposure temperature of the wood within the tree after a wildfire. The authors managed to relate the information obtained with solid-state nuclear magnetic resonance (ssNMR) of thermally degraded wood to the temperature the wood had been exposed to. This non-destructive method allows for the determination of the exposure temperature *after* the fire has occurred from a small core sample obtained from individual trees.

### Wildfires

The severity of a wildfire is determined by how fast the fire is spreading and at which temperature it burns. Wildfires occur in the form of crown, surface or ground fires—with decreasing intensity. According to Michaletz and Johnson^5^ maximum flame temperatures of wildfires are generally between 800 °C and 1000 °C, however, adiabatic temperatures can push this to 1325 °C.

However, even if the fire type is known, it is difficult to estimate the temperature and time of exposure. Research suggests that flame height—the distance from the base of the flame to the highest point^6^—and the speed of the fire can be used to form an estimate for the exposure temperature^7^. However, obtaining accurate measurements for flame height and speed can be challenging.

### Effect of thermal degradation on wood properties

The structural integrity of the wood often remains intact because the bark insulates against the high temperatures. Bark thickness and structure have a positively proportional relationship to cambium protection^8,9^ and cambium death occurs at temperatures around 60 °C^10^. Generally, wood is a poor heat conductor, while water has a much higher heat conductivity, which means that in trees with high MC the heat damage will reach further into the tree. On the other hand, the charred bark acts as heat insulator which protects the wood behind the bark.

Extractives, such as long-chain fatty acids begin to degrade between 150 °C and 200 °C, however the simultaneous removal of moisture may lead initially to a higher density than that of green wood, as the cell wall thickness of the wood increased due to compaction^11^.

Apart from extractives, hemicelluloses are the least thermally stable component of wood. They consist of low molecular weight sugars, which are less thermally stable than high molecular weight cellulose and their breakdown results in the formation of furfural, furan and mucic acid. At the same time the breakdown of acetyl groups results in the formation of acetic acid, which can catalyse further degradation^12^ These degradation products vaporize and exit the wood, resulting in significant mass loss that can be determined with thermal gravimetric analysis (TGA).

Temperatures exceeding 220 °C cause material loss due to the formation of volatile components and above 150 °C the chemical and physical properties of wood are permanently changed. As the temperature increases to 200–300 °C both hemicellulose and lignin start to degrade, where lignin commonly degrades at a slower rate and over a wider temperature range^13^.

Cellulose in the cell wall can be amorphous, which begins to degrade at 170 °C, or crystalline, which begins to degrade at 230 °C^14^. The mass loss of amorphous cellulose is generally larger than that of the crystalline cellulose. The mass loss increases with increasing exposure temperature and substantial mass loss occurs between 250 and 290 °C for amorphous cellulose and at 270–300 °C for crystalline cellulose. Above 300 °C, both types of cellulose are significantly degraded.

Van Groeningen et al.^11^ quantified the heat conductivity into the tree for *E. dunnii* and *E. macarthurii* and determined the temperatures of wood 1, 2 and 3 cm behind the bark for known exposure temperatures that were extrapolated to higher temperatures. Knowing the temperature to which the wood in the tree has been exposed, allows an informed choice if the wood is still suitable for structural purposes, pulping, or any other end use.

The variation in wood and bark structure, as well as MC of the tree makes it impossible to predict the exposure temperature of the wood based on species characteristics alone. We therefore propose a way to determine the temperature to which the *wood* was exposed from a small core sample extracted from directly behind the bark.

### Effect of wildfires on the forest industry

The forest industry is suffering from the increasing occurrence of wildfires as the degradation of the wood in burnt trees translates to huge economic losses. Financial impacts on both forestry and the wood products industry include timber volume loss, costs of unplanned harvesting and transport to salvage the wood, cost of replanting, disruption caused by unplanned age-class distributions, lost intake at sawmills and the value of lost production.

South Africa, for example, lost over 80,000 ha of forest plantations in 2007, which equates to R 250 million in timber volume loss alone, excluding damages to agriculture, human settlements, public works, and municipal infrastructures. The forestry industry needs to salvage any usable wood where possible, especially as fire mitigation becomes more difficult to control for plantation forestry globally.

The exposure to high temperatures leads to unknown wood degradation in the trees, which makes it difficult to determine which trees are still usable and which should be discarded. However, without the knowledge of the exposure temperature that the trees encountered, it is impossible to decide if the wood can still be used as intended. In South Africa it is often preferred to err on the safe side and discard the wood or use it for non-structural purposes only. The pulp industry will not use burnt wood at all, to avoid the char tainting the pulp.

### NMR analysis of wood

NMR spectroscopy is an analytical tool often used to identify the molecular composition of a sample by determining the response of different molecular components to a change in magnetic field. This technique is widely used throughout the chemical and pharmaceutical industries due to is accuracy and sophistication in determining molecular structures and anomalies. Proton NMR focuses on the nuclei of hydrogen atoms^15^. A signal is obtained by sending strong radio frequencies through a sample, which disturbs the magnetic moment of the protons and water molecules within the wood structure are an ideal target for the NMR technique. In the medical field the NMR technology is used to create 3D images of the magnetic moments of different molecules, which allows the easy distinction of different tissue types—this technique is called magnetic resonance imaging (MRI).

Wood is porous in nature and exchanges moisture with the surrounding environment. The moisture content (MC) of wood and the type of water that is present, i.e. bound, or free water heavily influences the NMR spectra. Numerous studies have explored the reason as to why the NMR signal broadens and relaxation times increase, when wood is subjected to thermal degradation^16–18^. These studies suggest that when wood is exposed to lower temperatures around ± 100 °C, the free water within the cells is removed without changing the bound water structure within the cell walls. This leads to a sharper peak and a narrow distribution of the relaxation time, as the decrease of MC to a certain point, shortens relaxation times. However, when temperatures increase to ≥ 250 °C the relaxation times become wider for White oak and Maple^16^. The authors propose that this is due to the unification of smaller pores into larger openings, resulting in a larger environment in which the free water is contained. Thermally modified Scots pine studied with NMR by Hietala et al.^17^ showed an increase of relaxation times with increasing temperatures, which was attributed to increasing porosity in the call wall structure.

The pilot study presented by Meincken and Berger^4^ analyzed the relationship between increasing exposure temperature and the NMR signal of the samples. The study determined a positive correlation between the HW and exposure temperature and two equations—a polynomial (parabola) and exponential curve—were found to best explain the correlation.

## Materials and methods

### Sample preparation

Sapwood samples of *Pinus patula* and *Eucalyptus grandis* were sourced from the Department of Forest and Wood Science, Stellenbosch, South Africa and cut into cubes of 3 mm side length with a bandsaw and subsequently stored in a conditioning room at 20 °C and 65% RH. These species were chosen, because they are the most widely used hard- and softwood species in South Africa. The wood used to develop the models was not exposed to wildfires, but exposed to well defined temperatures in a laboratory furnace. Only clear wood was used with a preference for earlywood in the sample cubes. Only sapwood was analysed, to represent the wood behind the bark in a standing tree.

After conditioning, the samples were exposed in triplicate to elevated temperatures in a Scientific Slab furnace 909-9 l for a fixed time of three minutes at 50, 70, 90, 110, 150, 200, 250 and 300 °C, respectively, forming a total of 24 samples.

Following thermal exposure, the samples were again stored in the conditioning room for 7 days to ensure that all changes due to thermal exposure were lasting and not only temporary after thermal exposure.

Although small cubes are not representative for the bulk of the tree trunk and thermal degradation will definitely happen at an elevated rate, due to the increase surface area, we found that they can be used do develop reasonable models and highlight the difference between hard—and softwoods. The sample size was limited by the sample chamber of the ssNMR.

### Validation samples

To verify the developed models, wood was sourced from hard- and softwood trees recently exposed to wildfires within South Africa. Small sapwood blocks were extracted from directly behind the bark. *Pinus patula* (age: 9 years) was sourced from York Timbers in Mpumalanga. *Eucalyptus grandis x nitens* (age: 5 years) was sourced from Sappi in Riverdale. The trees used to obtain validation samples were somewhat younger than the typical rotation age for these species, which means that wood was younger than the wood used to develop the models. This may have added to the uncertainty of the models.

The sample blocks were debarked, brushed and sanded to clean any remaining charcoal or ash that may affect the analysis. After cleaning, the blocks were cut to the appropriate sizes of 3 mm side length as close as possible to the bark side, as this part of the sapwood experienced the highest temperatures.

### NMR analysis

The ssNMR analysis was performed on a Bruker AvanceIII HD Ascend standard bore 500 MHz spectrometer (Bruker, Ettlingen, Germany) at the University of the Western Cape, South Africa.

Wide-line static ^1^H NMR spectra were acquired using a standard one-pulse pulse program with a 4 mm MAS BB NMR probe. The ^1^H π/2 pulse length was optimized at 4.0 μs with a recycle time of 4 s. The acquisition mode was set to qsim, the sweep width to 1600 ppm and the number of scans acquired were 16. The samples were packed into 4.0 mm zirconia rotors.

### Data analysis

The raw data contains the signal intensity as a function of frequency. All frequency curves were first base line corrected in Microsoft Excel before they were individually plotted in OriginLab for signal analysis, i.e. to determine peak area (PA) and half-width (HW) of the peaks.

Subsequently, the average value and standard error of the PA and HW were calculated for each exposure temperature and species and plotted as a function of exposure temperature. For better analysis the plots were also separated into a low and high temperature range from 0–150 to 150–300 °C.

Regression analysis was performed in OriginLab to obtain best fit functions and the R^2^ and χ^2^ values were determined for all equations.

The best performing models were chosen to estimate the exposure temperature of the validation samples.

### Statistical analysis

A Kruskal–Wallis non-parametric test and Dunn’s multiple comparisons method were conducted to determine if there was a statistically significant difference between the HW and PA values determined for the different exposure temperatures at a significance level of α = 0.1, as the sample values did not follow a normal distribution. Statistical analysis was performed using OriginLab software. Significantly different values are labelled with different letters.

### Validation experiments

The chosen models were resolved for *x* to be able to calculate the exposure temperature from the measured HW/PA value.

## Results and discussion

The average HW and PA of the NMR signals was plotted with error bars depicting the standard error as a function of the exposure temperature. All plots were subsequently fitted with various fit functions to obtain a suitable model to correlate the NMR signal with the exposure temperature.

**Figure 1:**
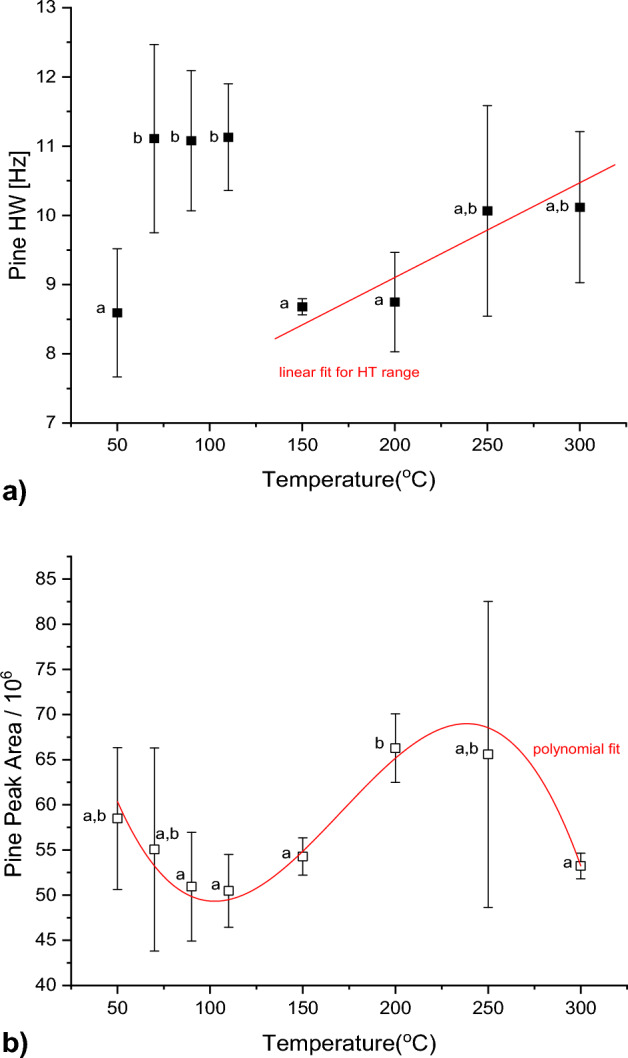
(**a**) HW signal and (**b**) PA of *Pinus patula* as a function of exposure temperature. Different letters indicate statistically different values. The best fit functions are indicated with a red line.

**Figure 2:**
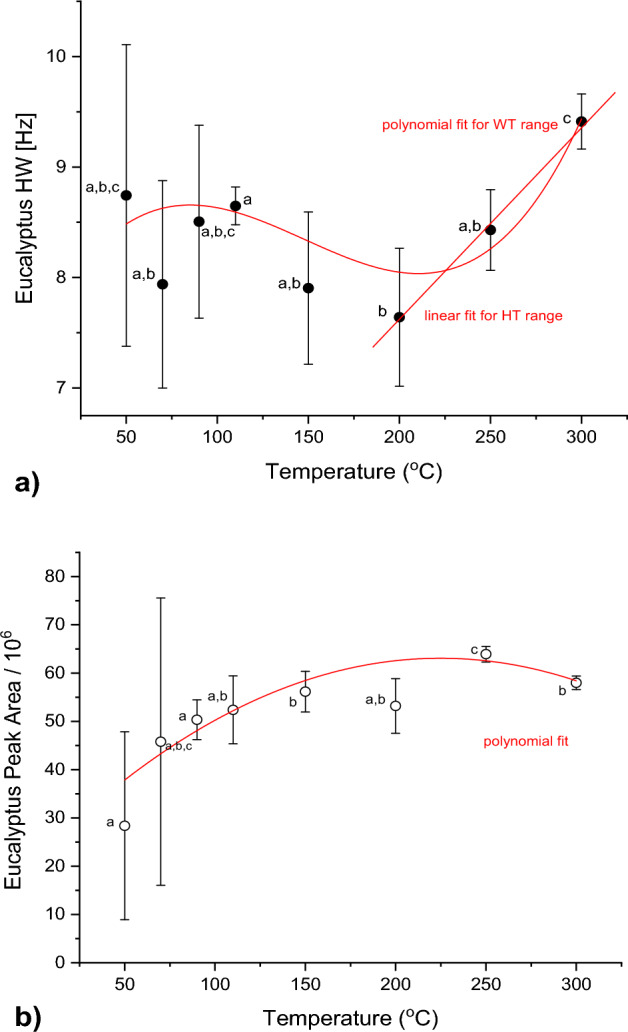
(**a**) HW signal and (**b**) PA of *Eucalyptus grandis* as a function of exposure temperature. Different letters indicate statistically different values. The best fit functions are indicated with a red line.

Figures 1 and 2 show the NMR results for Pine and Eucalyptus, respectively. The HW and PA over the entire temperature range was larger for Pine than Eucalyptus. The average HW of Pine was 9.94 Hz with a peak area of ± 56 × 10^6^, compared to an average HW of 8.40 Hz and peak area of ± 51 × 10^6^ for Eucalyptus. Standard errors were very large for both species, likely due to the small sample numbers, which was dictated by the experimental cost.

Figure 1a shows a steady increase of the HW with exposure temperature between 50 and 110 °C, followed by a sharp decline towards 150 °C and a subsequent increase towards 300 °C. 50, 150 and 200 °C share similar mean values for increasing temperature, and the mean HW values in the range of 50–110 °C are significantly different and may be regarded as outliers. The mean value increases significantly only for higher temperatures above 250 °C.

The PA (Fig. 1b) shows a gradual decrease with increasing exposure temperature until about 120 °C, after which it increases again with a maximum at around 230 °C.

There were distinct differences between the low and high temperature ranges, particularly 90–150 °C and 200 °C. Beyond 200 °C, the PA decreases towards 300 °C, where there is a significant difference between the mean values of 200 and 300 °C.

The HW of Eucalyptus is plotted in Fig. 2a. The values decrease somewhat between 50 and 200 °C and then increase rapidly towards 300 °C, in a similar trend found for Pine.

However, the variation between the HW values was larger for Eucalyptus, which can be seen in the larger error bars. Even though 110, 200 and 300 °C are significantly different, the means between exposure temperature groups can be regarded as equal. The large error in the temperature range of 50–90 °C could have probably been improved by using more samples or a more sensitive instrument.

The PA of Eucalyptus (Fig. 2b) increases consistently from 50 to 175 °C and then levels out. Significant differences were found between the exposure temperatures of 50–150 °C and the PA shows a positively proportional relationship to the exposure temperature, which was also found in a previous study by Meincken and Berger^4^.

When comparing the results from Pine and Eucalyptus no easily recognizable pattern exists between the HW, or PA and the exposure temperature, due to the large error bars and neither HW nor PA showed any obvious dominance in being the better prediction variable for exposure temperature.

All NMR signal plots were subsequently divided into a low (LT) and high (HT) temperature range between 0–150 °C and 150–300 °C, to obtain clearer relationships between the exposure temperature and the NMR signal that can be fitted.

The Pine peak area and Eucalyptus HW (LT) plots were deemed unsuitable for fitting, as the statistical analysis suggested high overlap of values and a random pattern. The high temperature range HW plots for both Pine and Eucalyptus (HT) were used to determine fit functions.

The Eucalyptus PA showed a strong positive proportional relationship for increasing exposure temperature for the whole range of temperature, which made it a strong candidate for fitting.

### Regression analysis

Various models were explored to fit the NMR signals as a function of temperature presented in Figs. 1 and 2. The whole temperature range (WT), LT and HT graphs were fitted with various functions and the fit equation, fit parameters and reduced χ^2^ value were determined.

**Figure 3:**
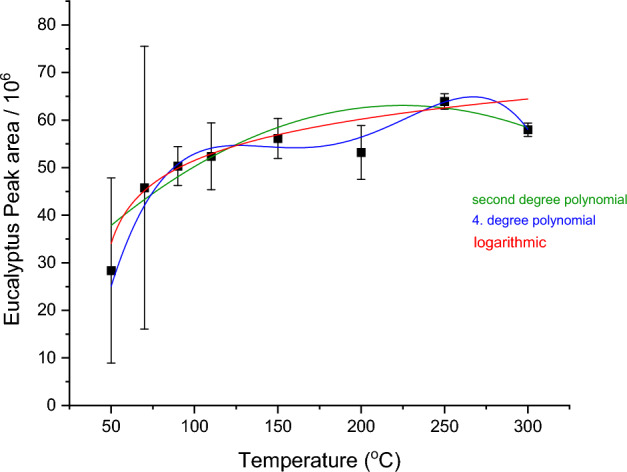
Examples of various regression curves for the PA of *E. grandis.*

Figure 3 shows the example of E. grandis with different regression curves fitted into the graph. The fit equations used for all graphs were, linear, polynomial up to fourth degree, exponential and logarithmic.

The χ^2^_ѵ_ value describes the variance between the chosen model and data. A good fit has a χ^2^ value of around 1, so 0.8–1.2 will be considered as a favorable range. χ^2^ > 1 implies that the model could be improved, or a more suitable model can be chosen. χ^2^ < 1 means that the model chosen was either too complex or errors are too big. These values were assessed to determine the most suitable fitting equations.

The best fit, as well as the simplest mathematical equations were chosen to describe the change of NMR signals with exposure temperature.

The best fit functions are summarized in Table 1, based on the simplicity of the equation and the estimated fit parameters for the chosen function describing as little change in the *x*-value or exposure temperature as possible.

**Table 1:** The best performing fit equations with acceptable χ^2^ values.

Equation #	Description	Equation	χ^2^
1	Pine HW (LT)	$y = A + Bx + Cx^{2}$	0.16
2	Pine HW (HT)	$y = A + Bx$	0.64
3	Eucalyptus HW (WT)	$y = A + Bx + Cx^{2} + Dx^{3}$	0.45
4	Eucalyptus HW (HT)	$y = A + Bx$	0.2
5	Eucalyptus PA (WT)	$y = A + Bx + Cx^{2}$	0.88

The best fitting function for Pine HW WT—a Gauss function—was disregarded as it was overly complicated.

### Validation testing

To validate the models, wood obtained from Pine and Eucalyptus trees that were subjected to a fairly recent wildfire, were analyzed with NMR and the HW and PA were determined.

The mean HW / PA value was calculated for each wood species to obtain an indication of the exposure temperatures of the fire, although individual trees may have been exposed to different temperatures.

The mean HW was 8.52 Hz for Pine and 6.69 Hz for Eucalyptus and the mean PA was 36.72 × 10^6^ for Pine and 51.22 × 10^6^ for Eucalyptus.

These values were then inserted into the chosen fit functions as *y* values and the equations were then solved for the *x*-value, in order to determine the exposure temperature. Table 2 lists the calculated exposure temperatures of the tested wood, determined from the functions from Table 1. Feasible solutions are printed in bold.

**Table 2:** The calculated exposure temperatures of the wood for different fit equations.

Equation #	Signal curve	Equation	T (°C)
1	Pine HW (LT)	$8.52 = 0.98 + 0.21x - 0.001x^{2}$	**x = 45.97**
2	Pine HW (HT)	$8.52 = 7.34 + 0.01x$	**x = 118**
3	Eucalyptus HW (WT)	$6.69 = 16.28 - 0.28 x + 0.003 x^{2} - 1.05 \times 10^{ - 5} x^{3}$	**x = 145.74**
4	Eucalyptus HW (HT)	$$6.69 = 5.36 + 0.01x $$	**x = 133**
5	Eucalyptus PA (WT)	$51.22 = 21.36 + 0.37 x - 8.26 \times 10^{ - 4} x^{2}$	**x = 105.60**

The linear, polynomial, and cubic functions produced two or more solutions for the exposure temperature, of which only one was in a realistic temperature range (below 150 °C) and was considered.

This temperature range was chosen based on the study by van Groeningen^11^, who measured the heat transfer into the tree 1, 2 and 3 cm underneath the bark of two *Eucalyptus* species and found that the wood directly behind the bark is exposed to elevated temperatures of around 125 °C for a fire temperature of about 1000 °C. Unfortunately, no similar model exists yet for the heat transfer into Pine trees, and this is part of ongoing studies. Unrealistic exposure temperatures were not considered.

According to anecdotal evidence, the fire at York Timber’s Pine plantation was severe with estimated temperatures of 1000 °C, which justifies the selection of exposure temperatures ≤ 150 °C. The wildfire in the Eucalyptus plantation from Sappi was somewhat milder with an estimated temperature of about 800–1000 °C, which justifies the selection of exposure temperatures up to 130 °C. Taking this into account, the linear function chosen from the Pine HT produces the best result with an exposure temperature of the wood behind the bark of 118 °C.

The Eucalyptus PA (WT) produced the most feasible result with small standard deviations above 70 °C, which made it the best candidate to predict exposure temperatures, with exposure temperatures of 106 °C.

The Eucalyptus HW HT yields an exposure temperature of 133 °C, which corresponds well with the reports of the wildfire. The wind was strong during the Sappi fire and some trees could have experienced more severe temperatures.

After considering all fit equations, the most adequate to estimate exposure temperature would be the linear functions for the HW in the high temperature region for Pine and Eucalyptus, and the parabolic function for the PA of Eucalyptus (WT). When taking all valid estimates into consideration, it appears that the Sappi plantation fire caused higher temperatures in the wood beneath the bark than the York plantation fire.

The wood exposure temperature can be used in conjunction with heat conductivity studies to estimate the fire temperature, which in the case of Eucalyptus can be estimated to have been between 550 °C and 800 °C. If one uses the Eucalyptus heat conductivity studies for Pine, then a minimum fire temperature of 700 °C can be estimated for the York wildfire.

The exposure temperature of the wood behind the bark allows sawmills and pulp mills to make an informed decision if the wood can still be used, or if it has been thermally degraded.

This project was the first step to determine the feasibility of estimating the temperature of a wildfire via the exposure temperature of the wood. We could show that the technique offers a valuable method to determine exposure temperatures non-destructively—and more importantly, retrospectively—from a small core sample obtained from burnt trees.

## Conclusions

This study explored whether the thermal degradation of wood due to wildfires could be estimated by determining the exposure temperature retrospectively with ssNMR. The results were promising, and models were developed to link the HW of the NMR signal to the exposure temperature for Pine and Eucalyptus.

For Pine, it was found that, similar to the pilot study, the HW signal had the best correlation to the exposure temperature and the best fit function was $y = A + Bx$.

For Eucalyptus the peak area showed a better correlation to the exposure temperature and the best fit function was $y = A + Bx + Cx^{2} .$

The linear functions for Eucalyptus and Pine HW HT produced worthy estimates, which was favoured as the data trends were similar.

Different models are needed for different wood species to estimate the exposure temperature.

This non-destructive method offers a new technique of determining the temperature of wildfires and the temperature to which the wood was exposed. Unlike other methods, which use fire intensity indices and other models, this method requires less variables to estimate the temperature and it does not require any measurements during the actual fire. Trees may be measured individually to determine the potential degradation that occurred within the stem, as wildfires do not affect every tree with the same severity within the stand.

The models could be improved with the use of larger sample sizes. A future study could concentrate on the best models derived from this study and increase the sample number to confirm the validity of the models. Extrapolation models of the temperature reached within the stem as a function of the distance from the bark should be developed for Pine, like those used for Eucalyptus trees^11^.

The frequency of wildfires around the world is continually increasing, as global temperatures increase. The forestry sector needs to prepare for this inevitable matter, and any further understanding with regards to the fire details such as intensity, or temperature of exposure will help in understanding the damage to the wood. Any wood that can be salvaged or repurposed must not be wasted, and with the knowledge of the exposure temperature this can be aided.

## Data Availability

All data used in this study is publicly available and can be obtained from the corresponding author on request.
